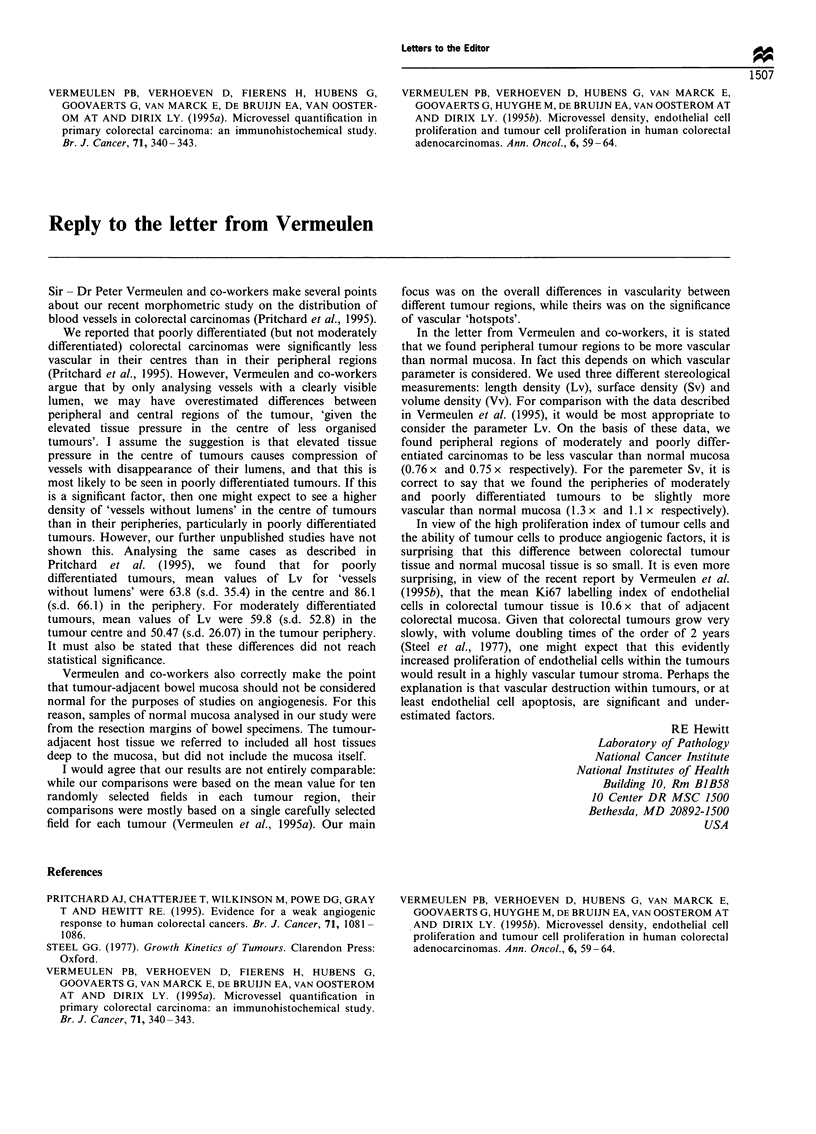# Reply to the letter from Vermeuler et al

**Published:** 1996-11

**Authors:** RE Hewitt


VERMEULEN PB, VERHOEVEN D, FIERENS H, HUBENS G, GOOVAERTS G, VAN MARCK E, DE BRUIJN EA, VAN OOSTEROM AT AND DIRIX LY. (1995*a*). Microvessel quantification in primary colorectal carcinoma: an immunohistochemical study. *Br. J. Cancer*, **71**, 340-343.

VERMEULEN PB, VERHOEVEN D, HUBENS G, VAN MARCK E, GOOVAERTS G, HUYGHE M, DE BRUIJN EA, VAN OOSTEROM AT AND DIRIX LY. (1995*b*). Microvessel density, endothelial cell proliferation and tumour cell proliferation in human colorectal adenocarcinomas. *Ann. Oncol.*, **6**, 59-64.

### Reply to the letter from Vermeulen

Sir – Dr Peter Vermeulen and co-workers make several points about our recent morphometric study on the distribution of blood vessels in colorectal carcinomas (Pritchard *et al.*, 1995).

We reported that poorly differentiated (but not moderately differentiated) colorectal carcinomas were significantly less vascular in their centres than in their peripheral regions (Pritchard *et al.*, 1995). However, Vermeulen and co-workers argue that by only analysing vessels with a clearly visible lumen, we may have overestimated differences between peripheral and central regions of the tumour, 'given the elevated tissue pressure in the centre of less organised tumours'. I assume the suggestion is that elevated tissue pressure in the centre of tumours causes compression of vessels with disappearance of their lumens, and that this is most likely to be seen in poorly differentiated tumours. If this is a significant factor, then one might expect to see a higher density of 'vessels without lumens' in the centre of tumours than in their peripheries, particularly in poorly differentiated tumours. However, our further unpublished studies have not shown this. Analysing the same cases as described in Pritchard *et al.* (1995), we found that for poorly differentiated tumours, mean values of Lv for 'vessels without lumens' were 63.8 (s.d. 35.4) in the centre and 86.1 (s.d. 66.1) in the periphery. For moderately differentiated tumours, mean values of Lv were 59.8 (s.d. 52.8) in the tumour centre and 50.47 (s.d. 26.07) in the tumour periphery. It must also be stated that these differences did not reach statistical significance.

Vermeulen and co-workers also correctly make the point that tumour-adjacent bowel mucosa should not be considered normal for the purposes of studies on angiogenesis. For this reason, samples of normal mucosa analysed in our study were from the resection margins of bowel specimens. The tumour-adjacent host tissue we referred to included all host tissues deep to the mucosa, but did not include the mucosa itself.

I would agree that our results are not entirely comparable: while our comparisons were based on the mean value for ten randomly selected fields in each tumour region, their comparisons were mostly based on a single carefully selected field for each tumour (Vermeulen *et al.*, 1995*a*). Our main focus was on the overall differences in vascularity between different tumour regions, while theirs was on the significance of vascular 'hotspots'.

In the letter from Vermeulen and co-workers, it is stated that we found peripheral tumour regions to be more vascular than normal mucosa. In fact this depends on which vascular parameter is considered. We used three different stereological measurements: length density (Lv), surface density (Sv) and volume density (Vv). For comparison with the data described in Vermeulen *et al.* (1995), it would be most appropriate to consider the parameter Lv. On the basis of these data, we found peripheral regions of moderately and poorly differentiated carcinomas to be less vascular than normal mucosa (0.76× and 0.75× respectively). For the paremeter Sv, it is correct to say that we found the peripheries of moderately and poorly differentiated tumours to be slightly more vascular than normal mucosa (1.3× and 1.1× respectively).

In view of the high proliferation index of tumour cells and the ability of tumour cells to produce angiogenic factors, it is surprising that this difference between colorectal tumour tissue and normal mucosal tissue is so small. It is even more surprising, in view of the recent report by Vermeulen *et al.* (1995*b*), that the mean Ki67 labelling index of endothelial cells in colorectal tumour tissue is 10.6× that of adjacent colorectal mucosa. Given that colorectal tumours grow very slowly, with volume doubling times of the order of 2 years (Steel *et al.*, 1977), one might expect that this evidently increased proliferation of endothelial cells within the tumours would result in a highly vascular tumour stroma. Perhaps the explanation is that vascular destruction within tumours, or at least endothelial cell apoptosis, are significant and underestimated factors.

RE Hewitt
*Laboratory of Pathology*
*National Cancer Institute*
*National Institutes of Health*
*Building 10, Rm B1B58*
*10 Center DR MSC 1500*
*Bethesda, MD 20892-1500*
*USA*

### References

PRITCHARD AJ, CHATTERJEE T, WILKINSON M, POWE DG, GRAY T AND HEWITT RE. (1995). Evidence for a weak angiogenic response to human colorectal cancers. *Br. J. Cancer*, **71**, 1081-1086.

STEEL GG. (1977). *Growth Kinetics of Tumours*. Clarendon Press: Oxford.